# c-Jun-dependent β3GnT8 promotes tumorigenesis and metastasis of hepatocellular carcinoma by inducing CD147 glycosylation and altering *N*-glycan patterns

**DOI:** 10.18632/oncotarget.24192

**Published:** 2018-01-12

**Authors:** Chunliang Liu, Hao Qiu, Dandan Lin, Zerong Wang, Ning Shi, Zengqi Tan, Jun Liu, Zhi Jiang, Shiliang Wu

**Affiliations:** ^1^ Department of Biochemistry and Molecular Biology, The First Affiliated Hospital of Soochow University, Soochow University, Suzhou, Jiangsu 215123, P.R. China; ^2^ Department of Infectious Diseases, The Fifth People's Hospital of Suzhou, Suzhou, Jiangsu 215007, P.R. China; ^3^ Department of Physiology and Pharmacology, University of Georgia, Athens 30602 GA, USA; ^4^ College of Life Science, Northwest University, Xian, Shanxi 710069, P.R. China

**Keywords:** β3GnT8, hepatocellular carcinoma, CD147, tumorigenesis, glycosylation

## Abstract

β3GnT8, a key polylactosamine synthase, plays a vital role in progression of various types of human cancer. The role of β3GnT8 in hepatocellular carcinoma (HCC) and the underlying mechanisms, however, remain largely unknown. In this study, we found that β3GnT8 and polylactosamine were highly expressed in HCC tissues compared with those in adjacent paracancer tissues. Overexpression of β3GnT8 promoted while knockdown of β3GnT8 inhibited HCC cell invasion and migration *in vitro*. Importantly, enhanced tumorigenesis was observed in nude mice inoculated with β3GnT8-overexpressing HCC cells, suggesting that β3GnT8 is important for HCC development *in vitro* and *in vivo*. Mechanistically, β3GnT8 modulated the N-glycosylation patterns of CD147 and altered the polylactosamine structures in HCC cells by physically interacting with CD147. In addition, our data showed the c-Jun could directly bind to the promoter of β3GnT8 gene and regulate β3GnT8 expression. β3GnT8 regulated HCC cell invasion and migration in a C-Jun-dependent manner. Collectively, our study identified β3GnT8 as a novel regulator for HCC invasion and tumorigenesis. Targeting β3GnT8 may be a potential therapeutic strategy against HCC.

## INTRODUCTION

Hepatocellular carcinoma (HCC), a primary malignancy of the liver arising from the hepatocytes, is one of the leading causes of cancer-related death worldwide. Despite the recent development of standard treatment options, including tumor resection, percutaneous radiofrequency ablation, chemotherapy, and liver transplantation, the morbidity and mortality related to HCC are rapidly increasing, posing a significant threat to human health [1, 2]. Thus, identifying new potential diagnostic markers and therapeutic targets is urgently required for early detection and effective treatment of HCC.

Protein glycosylation is a posttranslational process enzymatically driven by glycosyltransferases (GTs) and glycosidases. Aberrant glycosylation of glycoproteins due to the alteration of expression of GTs is associated with carcinogenesis and also plays an important role in cancer invasion and metastasis, thus being considered a potential biomarker for cancers [3, 4]. For example, high expression of β1,6-*N*-acetylglucosaminyltransferase (GnT) that catalyzes the formation of β1,6-branching of *N*-linked glycans on membrane proteins is correlated with tumor metastasis and is a key event in the early stage of hepatocarcinogenesis [5]. Branched *N*-glycans are further elongated via the addition of poly-*N*-acetyllactosamine (polylactosamine) by β1,3-*N*-acetylglucosaminyltransferases (β3GnTs) [6].

β3GnT8, a key polylactosamine synthase originally termed β3GalT7 [7], was first cloned by our laboratory and another research group [6]. Our previous study has demonstrated that β3GnT8 is able to promote the metastasis of colorectal cancer by catalyzing the elongation of β1,6-branched polylactosamines of cluster of differentiation 147 (CD147), a transmembrane glycoprotein mediating tumor invasion and metastasis [8, 9]. Blockade of CD147 by specific antibody or knockdown of CD147 could suppress the growth and metastasis of HCC cells *in vitro* [10], suggesting that CD147 is a potential therapeutic target for HCC. *N*-glycosylation of CD147 yields highly glycosylated (HG)-CD147 majorly carrying 1,6-branched polylactosamines [11], and has been demonstrated to be instrumental in promoting malignant transformation [12], whereas deglycosylated CD147 fails to mediate tumor metastasis [13]. However, the regulation of glycosylation of CD147 in HCC is not fully understood.

In the present study, we demonstrated that both β3GnT8 and polylactosamines were overexpressed in HCC tissues compared with the adjacent paracancer tissues, and investigated the role of β3GnT8 in the metastatic potential of HCC *in vitro* and tumorigenesis *in vivo* by gain- and loss-of-function assays. Importantly, we found that β3GnT8 is responsible for glycosylation of CD147 in HCC. The mechanism underlying the regulation of β3GnT8 expression was also elucidated.

## RESULTS

### β3GnT8 and polylactosamine are overexpressed in HCC tissues

**Figure 1:**
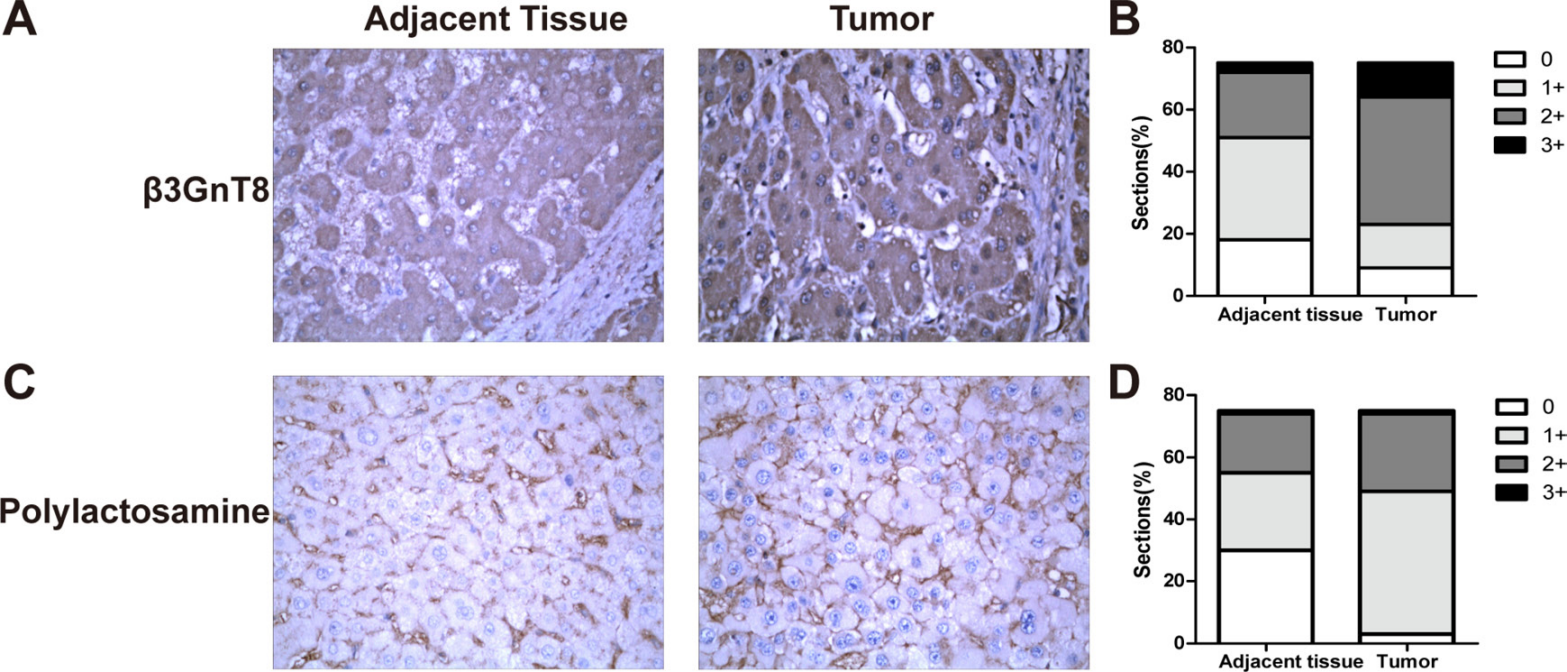
β3GnT8 expression in HCC cell lines and tissues IHC and LEL staining for β3GnT8 (**A** and **B**) polylactosamines (**C** and **D**) in HCC tissues and the adjacent paracancer tissues, respectively, which was quantified according to the percentage of positive cells to total cells. Magnification, ×400.

To investigate the role of β3GnT8 in HCC, we examined the protein expression of β3GnT8 in HCC tissues and the paracancer tissues. As shown in Figure 1A, β3GnT8, mainly localized in the cytoplasm of cells, was moderately or highly expressed in 69.3% (52/75) of HCC tissues compared with the adjacent paracancer tissues (Figure 1B). The mean IHC score of β3GnT8 in 75 HCC tissues was significantly higher than that in the adjacent paracancer tissues (1.72 *vs* 1.12; *P* < 0.001). Because β3GnT8 catalyzes the biosynthesis of polylactosamine chains, we also determined the tissue levels of polylactosamines using LEL staining [14]. We found that polylactosamines were predominantly localized in the membrane and cytoplasm of cells and the levels of polylactosamines were higher in HCC tissues than those in the adjacent paracancer tissues (Figure 1C and 1D). Taken together, these results suggest that the expression of β3GnT8, as well as the levels of polylactosamines, was significantly upregulated in HCC tissues and may serve as a diagnostic biomarker for HCC.

### β3GnT8 promotes HCC invasion and migration *in vitro* as well as tumorigenesis *in vivo*

**Figure 2:**
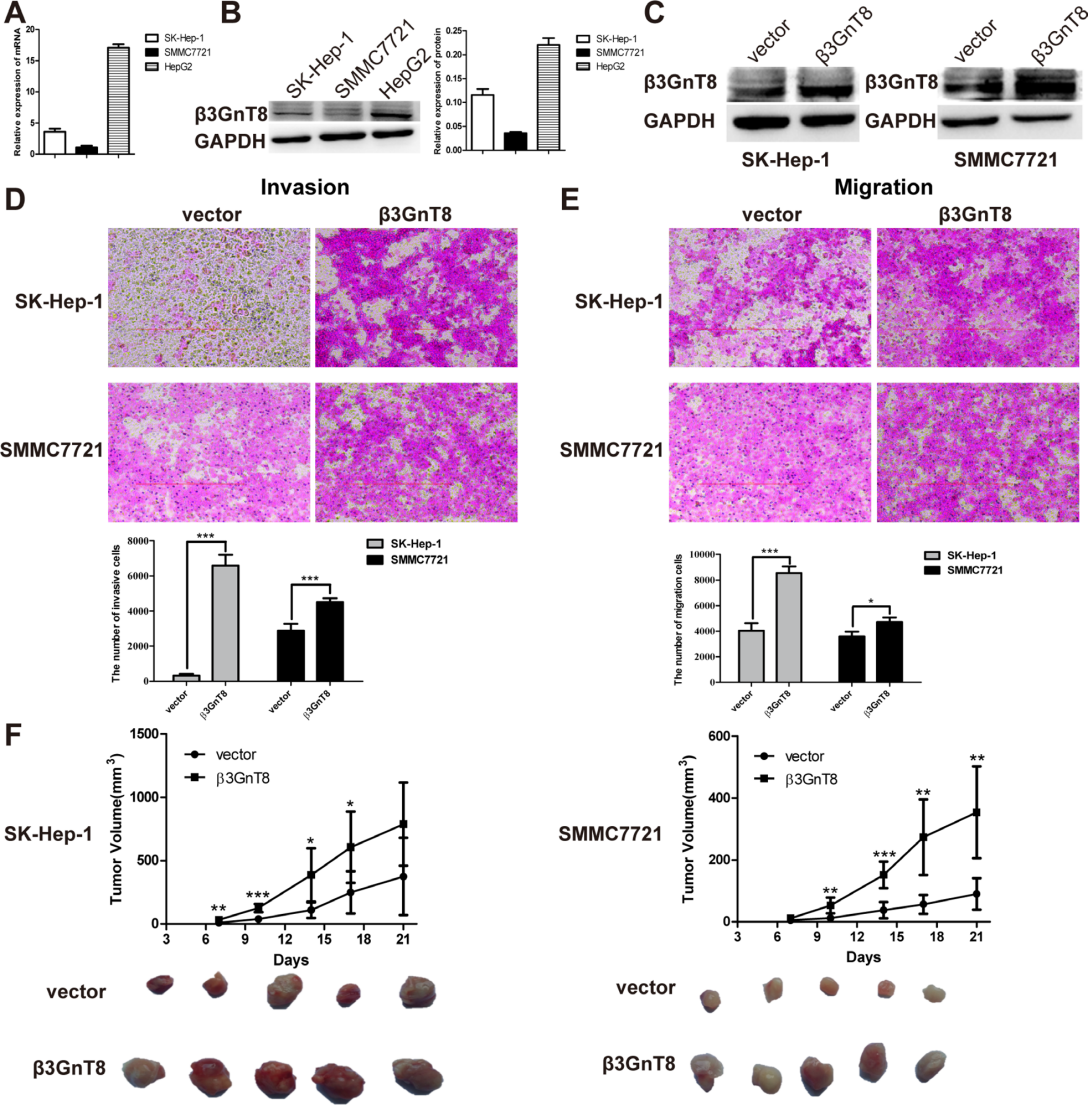
The effects of β3GnT8 on HCC migration and invasion *in vitro* and tumorigenesis *in vivo* (**A**) qPCR detection of β3GnT8mRNA expression in HCC cell lines. (**B**) Western blot detection of β3GnT8 protein expression in HCC cell lines. (**C**) Western blot assay was performed to confirm the ectopic expression of β3GnT8 in stably transfected SK-Hep-1 and SMMC7721 cells. Transwell assays were performed to assess the invasion (**D**) and migration (**E**) abilities of β3GnT8-overexprssing SK-Hep-1 (upper panel) and SMMC7721 (lower panel) cells. The representative images are shown. Magnification, ×100. (**F**) Tumor growth curves of nude mice inoculated with β3GnT8-overexprssing HCC cells (upper panel) and representative photographs of primary tumors derived from β3GnT8-overexprssing HCC cells (lower panel). Data are representative of three independent experiments and presented as means ± SD; ^*^p < 0.05, ^**^p < 0.01, ^***^p < 0.001.

We next sought to examine the expression of β3GnT8 *in vitro*, using three HCC cell lines SK-Hep-1, SMMC7721 and HepG2. As shown in Figure 2A and 2B, both mRNA and protein expression of β3GnT8 was much stronger in HepG2 cells than that in SK-Hep-1 and SMMC7721 cells. These cells were then used for the following loss- or gain-of-function assays, respectively.

To investigate the function of β3GnT8 in HCC metastasis *in vitro*, we performed transwell invasion and migration assays in SK-Hep-1 and SMMC7721 cells with stable expression of β3GnT8. The overexpression of β3GnT8 was confirmed using western blot analysis (Figure 2C). As shown in Figure 2D and 2E, the numbers of invading or migrating cells overexpressing β3GnT8 were dramatically increased compared with those in the control groups for both cell lines. We also detect the expression of epithelial mesenchymal transitions marker, while only the expression of β-catenin was increased slightly in β3GnT8 overexpression cells (Supplementary Figure 1A and 1B). To further investigate the effect of β3GnT8 on tumorigenesis *in vivo*, SK-Hep-1 or SMMC7721 cells with stable expression of β3GnT8 or control vector were subcutaneously inoculated into the nude mice. As shown in Figure 2F, mice inoculated with β3GnT8-expressing vectors exhibited a marked increase in tumor size in a time-dependent manner, compared with the control groups. And the expression of Ki-67, as a proliferation maker, was also increased in β3GnT8-expressing cells (Supplementary Figure 1C and 1D). These data suggest that β3GnT8 is sufficient to promote HCC metastasis and tumorigenesis *in vitro* and *in vivo*, respectively.

### Knockdown of β3GnT8 suppresses HCC invasion and migration *in vitro*

**Figure 3:**
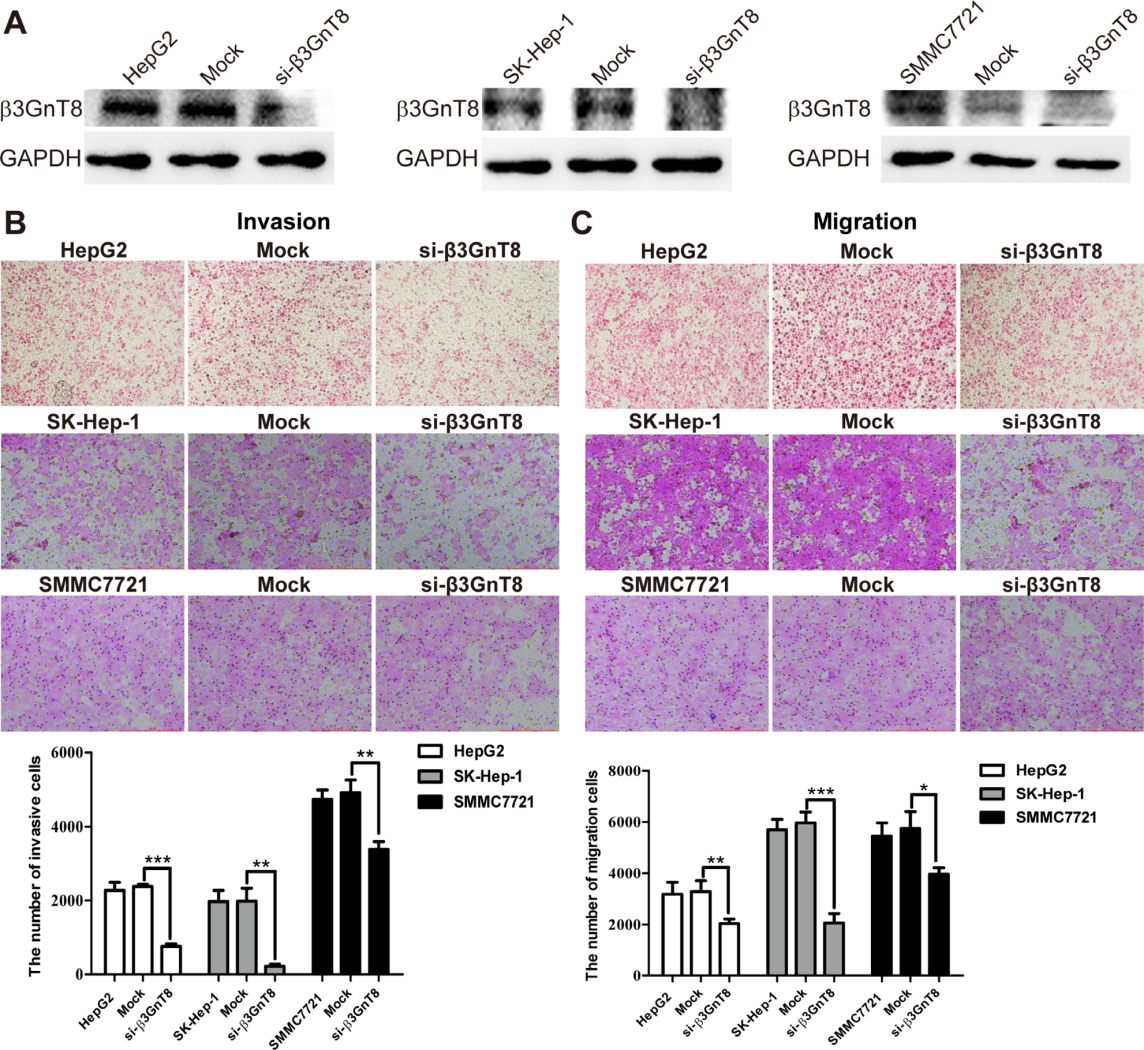
The effects of β3GnT8 knockdown on HCC invasion and migration *in vitro* (**A**) Western blot assay was performed to confirm knockdown of β3GnT8 in HCC cells. Transwell assays were performed to assess the invasion (**B**) and migration (**C**) abilities of β3GnT8-silenced HCC cells. Data are representative of three independent experiments and presented as means ± SD; ^*^p < 0.05, ^**^p < 0.01, ^***^p < 0.001.

To explore whether β3GnT8 is required for invasion and migration of HCC cells *in vitro*, we knocked down the endogenous expression of β3GnT8 in SK-Hep-1, SMMC7721 and HepG2 cell lines using pSilencircle-si-β3GnT8 plasmids, which was confirmed by Western blot analysis (Figure 3A). As shown in Figure 3B and 3C, in all the three cell lines, knockdown of β3GnT8 significantly decreased the numbers of invading or migrating cells compared with the control groups, suggesting that β3GnT8 is critically essential for the metastatic potential of HCC cells *in vitro*.

### β3GnT8 regulates intercellular level of polylactosamines and alters the glycopattern in HCC cells

**Figure 4:**
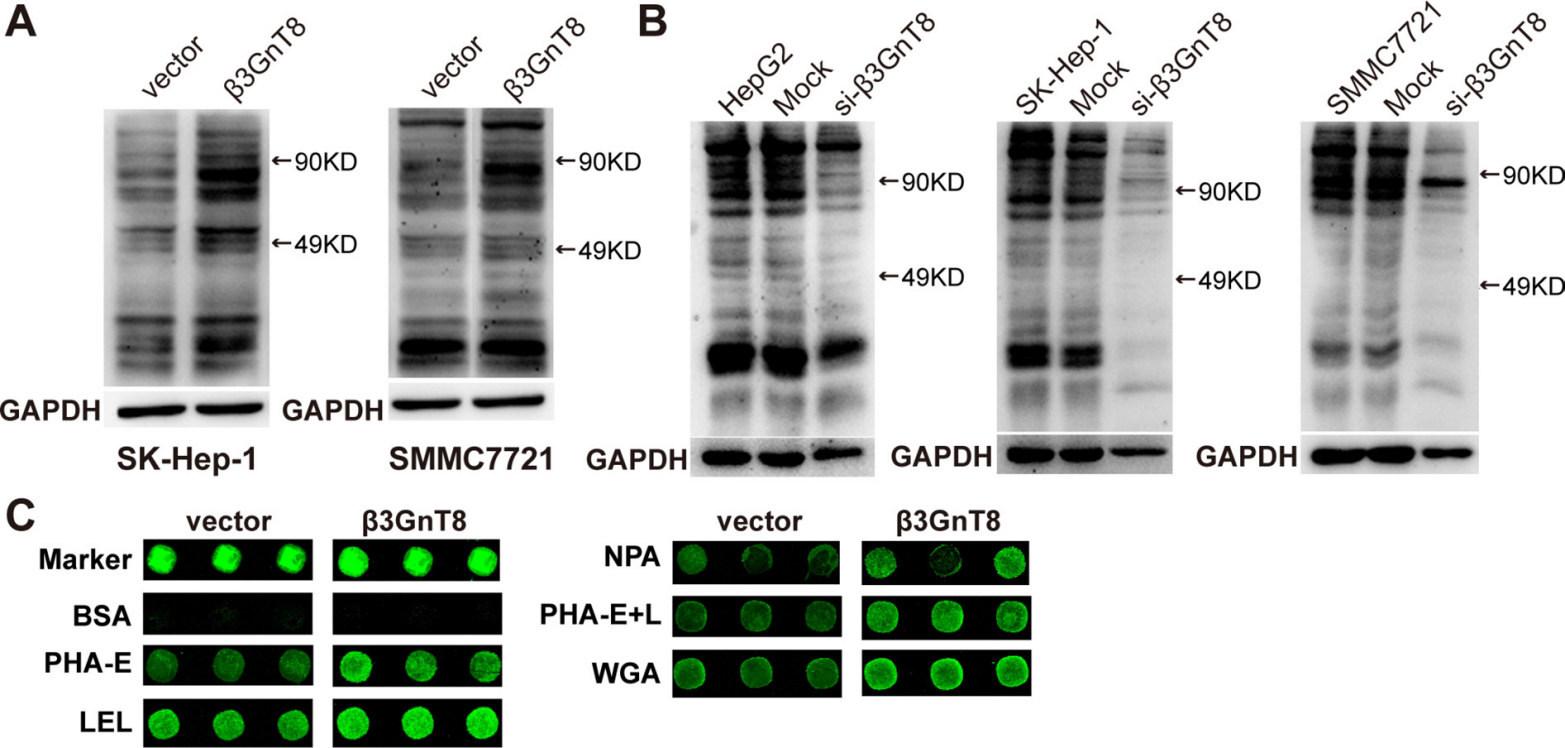
The effect of β3GnT8 on the expression of polylactosamines in HCC cells The protein samples obtained from cells were separated by SDS-PAGE and transferred to nitrocellulose membranes which were incubated with biotinylated-LEL for detection of polylactosamines expression. (**A**) Lectin blot analysis of polylactosamines in β3GnT8-overexpressing HCC cells. (**B**) Lectin blot analysis of polylactosamines in β3GnT8-silenced HCC cells. (**C**) Fluorescent images of lectin microarrays for β3GnT8-overexpressing SK-Hep-1 cells were shown. Each lectin (green) was detected in triplicates.

Since aberrant *N*-linked β1,6-branching of polylactosamines contributes to cancer progression [15], we next performed a lectin blot assay with biotinylated-LEL to explore whether β3GnT8 has effects on the synthesis of total polylactosamines in HCC cells. The results showed that overexpression of β3GnT8 increased while knockdown of β3GnT8 reduced the levels of polylactosamines in HCC cells (Figure 4A and 4B). To further examine the effect of β3GnT8 on the glycopattern, which reflects the expression, function, and metabolism of oligosaccharides in cells, we performed a lectin microarray assay containing 37 lectins to analyze the differential expression of glycans on SK-Hep-1 cells. As shown in Figure 4C and Table 1, the levels of five glycans recognized by lectins PHA-E, LEL, NPA, PHA-E+L and WGA were found significantly upregulated in the cells in response to β3GnT8 overexpression. These data suggest that β3GnT8 may contribute to the regulation of the intercellular levels of polylactosamines as well as the glycopattern in HCC cells.

**Table 1:** Differential lectins bound to β3GnT8 group compared to the vector control group by the lectin microarray analysis

Lectin	Specificity	Change
PHA-E	Bisecting GlcNAc, biantennary complex-type N-glycan with outer Gal	↑46.50%
LEL	(GlcNAc)n, high mannose-type N-glycans	↑15.70%
NPA	High-Mannose, Manα1-6Man	↑139.50%
PHA-E+L	Bisecting GlcNAc, bi-antennary N-glycans, tri- and tetra-antennary complex-type N-glycan	↑60.40%
WGA	Multivalent Sia and (GlcNAc)n	↑50.00%

### β3GnT8 regulates HG-CD147 via a physical interaction

**Figure 5:**
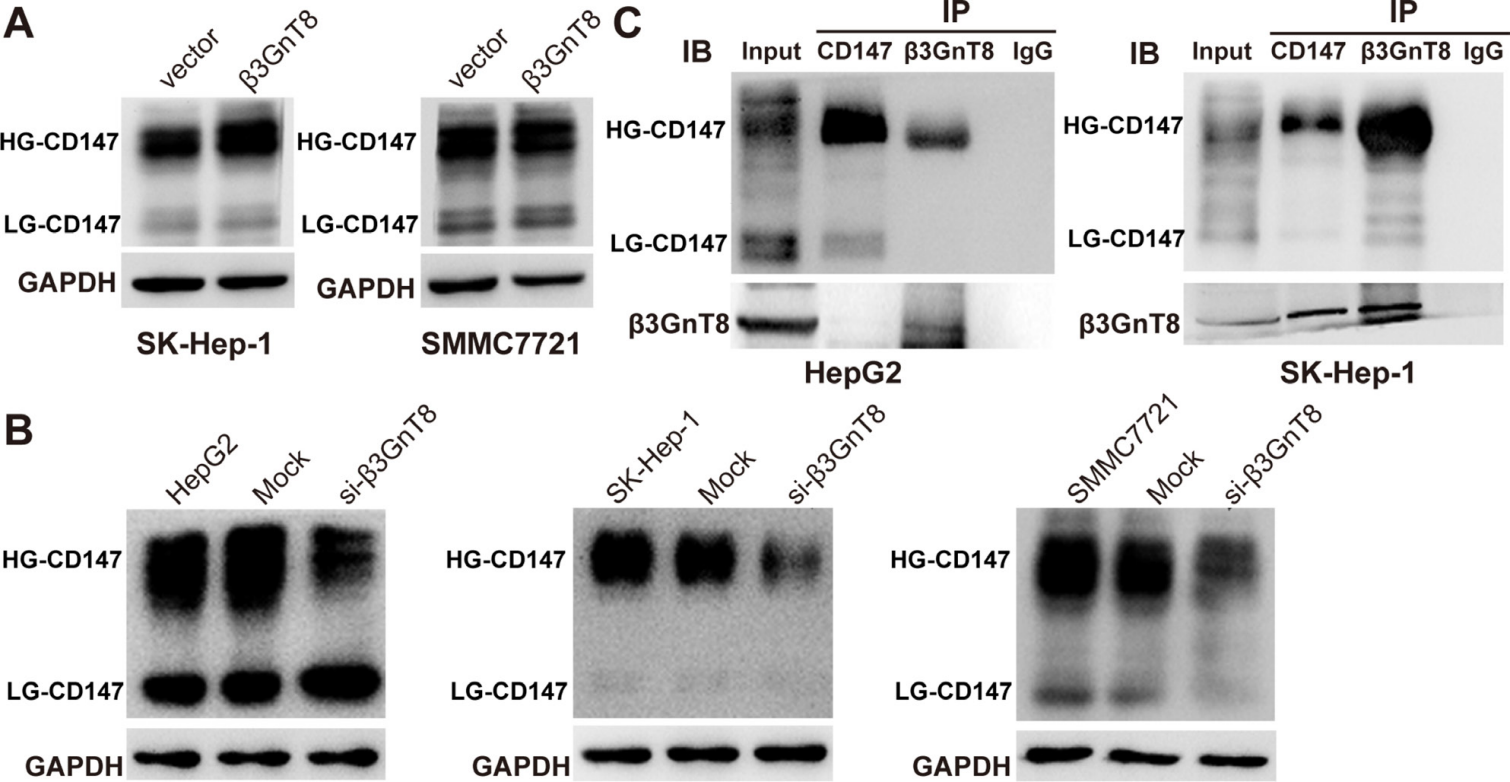
The effect of β3GnT8 on glycosylation of CD147 in HCC cells (**A**) Western blot analysis of highly glycosylated (HD)-CD147 and lowly glycosylated (LD)-CD147 in β3GnT8-overexpressing HCC cells. (**B**) Western blot analysis of HD-CD147 and LD-CD147 in β3GnT8-silenced HCC cells. (**C**) Co-IP assay for detection of interaction between β3GnT8 and CD147. IgG was used as blank control.

β3GnT8 is able to catalyze the elongation of β1,6-branched polylactosamines of CD147 (HG-CD147) which promotes the metastasis of colorectal cancer. And glycoprotein CD147 is a cancer-associated biomarker and HG-CD147 can promote tumor metastasis in HCC [16, 17], we next sought to investigate whether β3GnT8 could affect CD147 glycosylation in HCC cells. As shown in Figure 5A, ectopic expression of β3GnT8 markedly elevated the levels of HG-CD147 in HCC cells in comparison with the controls, but appeared to have no effects on the levels of LG-CD147, although HG-CD147 had a much stronger expression than LG-CD147 in a basal state. On the other hand, knockdown of β3GnT8 significantly decreased the levels of HG-CD147 in all the three HCC cell lines (Figure 5B). These results demonstrate that HG-CD147 is functionally dependent on β3GnT8, suggesting a possible physical interaction between these two molecules. The mechanism of β3GnT8 affect CD147 expression, directly or indirectly, is still unclear. To test the interaction between β3GnT8 and CD147, Co-IP assay was performed using endogenous proteins extracted from HepG2 and SK-Hep-1 cells to ensure whether β3GnT8 regulates of CD147 through binding to CD147 protein directly. As shown in Figure 5C, a substantial amount of HG-CD147 was co-immunoprecipitated with β3GnT8 in both cell lines. In addition, β3GnT8 was also co-immunoprecipitated with HG-CD147 in SK-Hep-1 cells, but to a lesser degree in HepG2 cells (Figure 5C). In contrast, a very weak interaction between β3GnT8 and LG-CD147 was observed in these two cell lines (Figure 5C). Collectively, these results show that β3GnT8 predominantly interacted with HG-CD147, but weakly interacted with LG-CD146 in HCC cells, suggesting a possible role of β3GnT8 in extensive glycosylation of CD147.

### β3GnT8 alters N-glycan patterns in HCC cells

**Figure 6:**
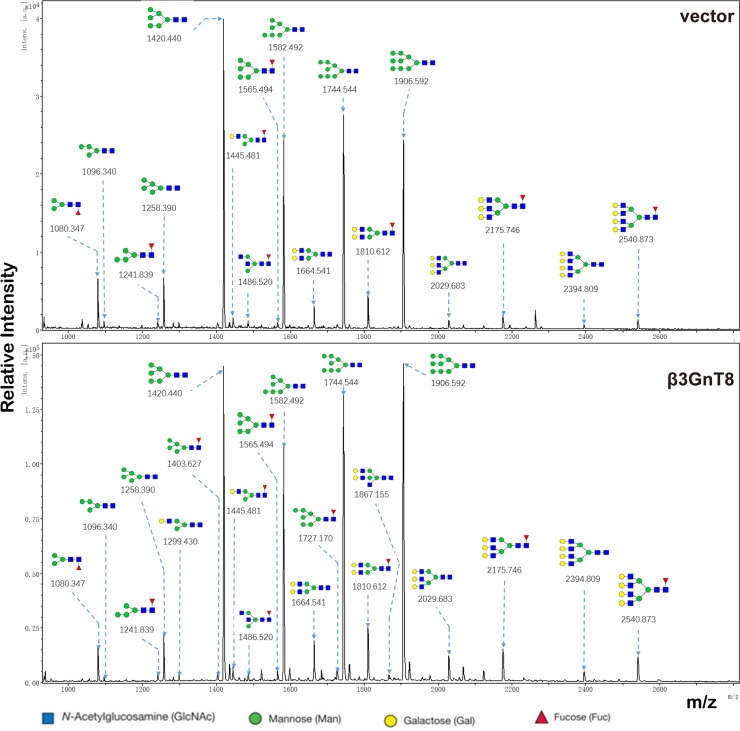
MALDI-TOF/TOF-MS/MS analysis of N-glycan precursor ions in MS spectra MS/MS analysis of precursor ions was performed and cleavages were obtained, including B, Y, C, and Z glycosidic cleavages and A and X cross-ring cleavages. Structures of cleavage ions and m/z values of Sk-Hep-1/vector and SK-Hep-1/β3GnT8 are shown.

We further examined the role of β3GnT8 in regulating *N*-glycosylation in SK-Hep-1 cells using a MALDI-TOF/TOF-MS analysis. Representative MALDI-TOF/TOF-MS spectra of N-glycans with signal-to-noise ratios > 3 from total glycoproteins were annotated using the GlycoWorkbench software program (Figure 6). We found that 17 out of a total of 21 N-glycan structures were present in both SK-Hep-1/vector and SK-Hep-1/β3GnT8 cells, and 4 unique ones were present in SK-Hep-1/β3GnT8 cells (m/z 1299.430, m/z 1403.627, m/z1727.170, m/z 1867.155) (Supplementary Table 1). Table 2 indicated the N-glycans profiles in SK-Hep-1/vector and SK-Hep-1/β3GnT8 cells. β3GnT8 overexpression led to an increase in percentages of the complex-type, hybrid, Bi-, tri-, and Bisecting GlcNAc, but not in percentage of high-mannose-type N-glycan structures. Expectedly, percentage of potential poly-LacNAc N-glycan structures was higher in SK-Hep-1/β3GnT8 cells (12.80%) than in control cells (9.07%), suggesting a key role of β3GnT8 in N-glycosylation in HCC cells.

**Table 2:** Relative variation of major types of N-glycans in SK-Hep-1/vector and SK-Hep-1/β3GnT8 cell lines

Glycan type	Relative Proportion(%)	
	vector	β3GnT8
High mannose	85.33%	83.12%
Complex	10.22%	13.55%
Hybrid	8.37%	11.05%
Bi-antennary	9.26%	12.4%
Tri-antennary	2.89%	4.87%
Tetra- and Penta-antennary	20.69%	22.66%
Bisecting GlcNAc	8.37%	11.04%
Fucosylation	12.58%	13.45%
Potential Ploy-LacNAc	9.07%	12.80%

### c-Jun regulates β3GnT8 expression through binding to the promoter of β3GnT8 gene

**Figure 7:**
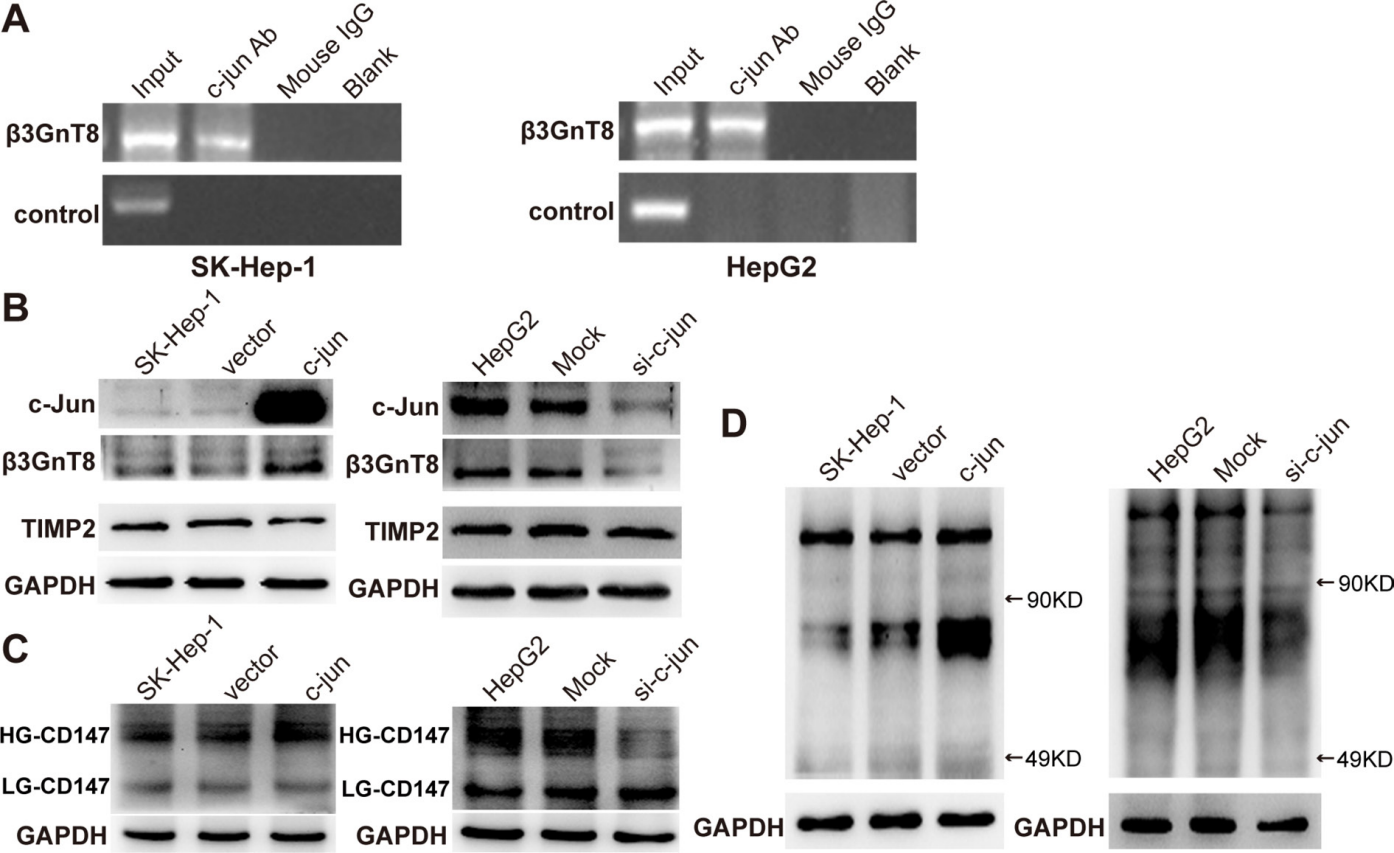
The effects of c-jun on the expression of β3GnT8, polylactosamines and glycosylation of CD147 in HCC cells (**A**) Chromatin DNAs was purified from SK-Hep-1 and HepG2 cells via anti-c-Jun or mouse IgG antibody, and then was subjected to PCR analysis to detect the binding of c-Jun to β3GnT8 promoter in SK-Hep-1 (left panel) and HepG2 (right panel) cells. Input, sonicated chromotin samples; c-jun Ab, immunoprecipitation with anti-c-jun antibody; mouse IgG, immunoprecipitation with mouse IgG; Blank, no immunoprecipitation. Western blot and lectin blot analysis was performed to determine the expression of β3GnT8, TIMP2 (**B**), HG-CD147, LG-CD147 (**C**), and polylactosamines (**D**) in SK-Hep-1 (left panel) and HepG2 (right panel) cells with c-jun overexpression and knockdown, respectively.

Since β3GnT8 contributes to N-glycosylation, HG-CD147, and subsequently tumorigenesis, we next sought to investigate the mechanism underlying β3GnT8 expression. c-Jun has been found to be an important transcription factor regulating glycosylation-related genes in cancer development [18], and our laboratory has demonstrated that −561/+8 is a potential c-jun binding sequence on the β3GnT8 promoter via a luciferase reporter system [19], which prompted us to hypothesize that β3GnT8 is a direct downstream target of c-Jun in HCC. To test this hypothesis, ChIP assay was performed using SK-Hep-1 and HepG2 cell lines. As shown in Figure 7A, the binding of c-Jun to promoter of β3GnT8 gene was detected in both cell lines, suggesting the interaction between c-Jun and promoter of β3GnT8 gene. Importantly, overexpression of c-Jun promoted while knockdown of c-Jun inhibited β3GnT8 expression in HCC cells (Figure 7B), suggesting that β3GnT8 is a downstream target of c-Jun. Consistently, the similar trend was also observed in expression levels of HG-CD147 and polylactosamines (Figure 7C and 7D), whereas tumor metastasis-related tissue inhibitor of metalloproteinases 2 (TIMP2) expression was inhibited by c-Jun (Figure 7B). These results suggest that c-Jun may function in HCC via regulation of β3GnT8.

**Figure 8:**
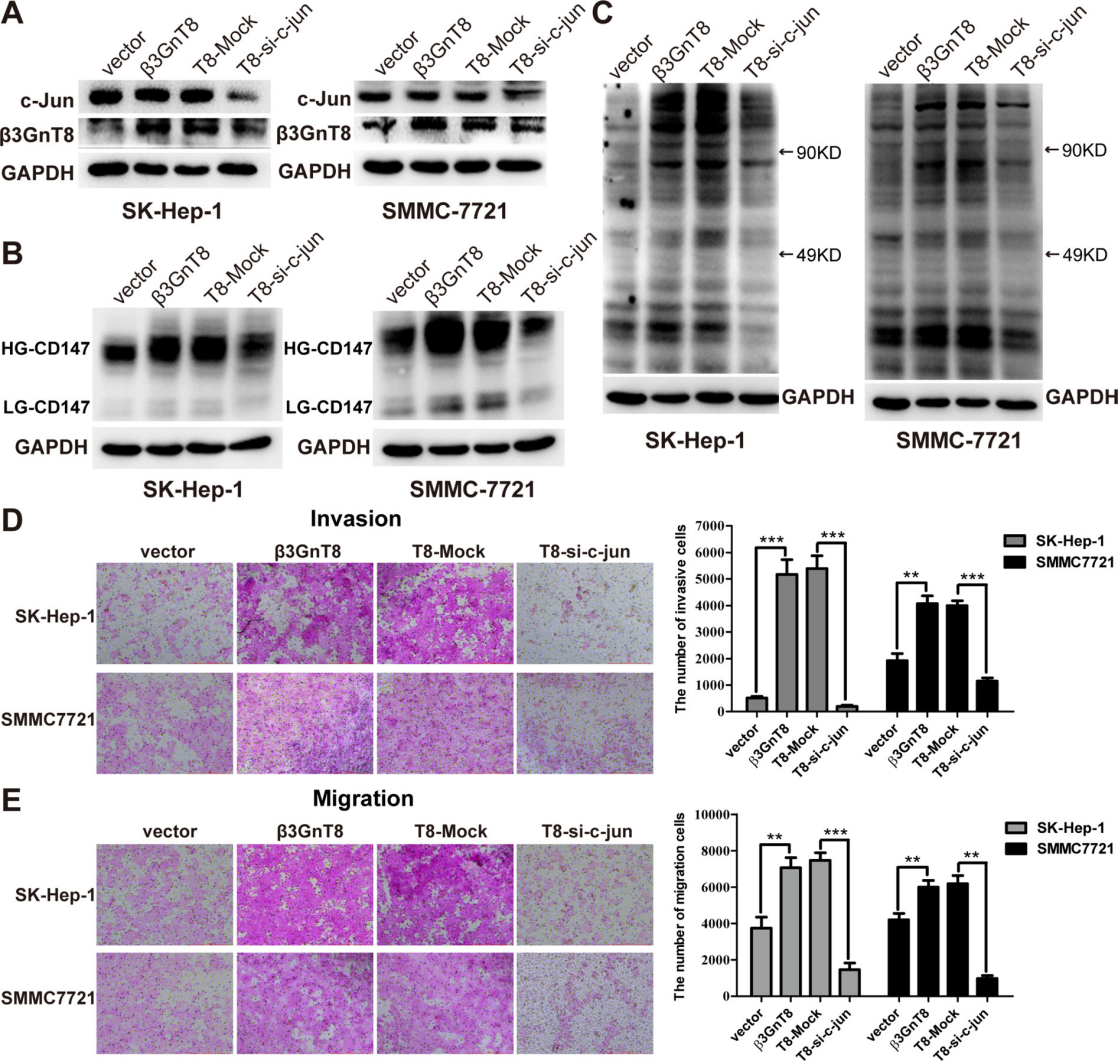
The function of β3GnT8 was regulated by c-Jun in HCC cells HCC cells were transfected with vector control (vector), β3GnT8 vector (β3GnT8), both β3GnT8 and mock control (T8-Mock), and, both β3GnT8 and si-c-jun (T8-si-c-jun). Western blot and lectin blot assays were performed to examine the expression of c-jun, β3GnT8 (**A**), HG-CD147, LG-CD147 (**B**), and polylactosamines (**C**). Transwell assays were performed to assess the invasion (D) and migration (E) abilities of HCC cells. Data are representative of three independent experiments and presented as means ± SD; ^**^p < 0.01, ^***^p < 0.001.

To further confirm the regulation of β3GnT8 by c-Jun, HCC cells were transfected with vector control and β3GnT8 vector, and β3GnT8-expressing cells were co-transfected with mock control and si-c-jun plasmids. Western blot analysis showed that β3GnT8 was overexpression in β3GnT8-expressing and T8-mock control cells, and knockdown of c-Jun decreased the expression of c-jun and β3GnT8 in T8-si-c-jun cells when compared with mock control cells (Figure 8A). As shown in Figure 8B and 8C, knockdown of c-Jun by si-c-Jun markedly suppressed β3GnT8-induced expression of HG-CD147 and polylactosamines as well as HCC cell invasion and migration by blocking β3GnT8 expression, indicating that expression and function of β3GnT8 are dependent on c-Jun. Taken together, these findings suggest that c-Jun regulates β3GnT8 expression through directly binding to the promoter of β3GnT8 gene, and subsequently regulate β3GnT8 functions in protein glycosylation and HCC metastasis.

## DISCUSSION

Our previous studies have revealed that high expression of β3GnT8 was correlated with various malignancies, such as cervix tumor, gastric cancer and glioma [20, 8, 21]. However, the role of β3GnT8 in HCC remains unclear. In the present study, we demonstrated for the first time that the expression of β3GnT8 was significantly upregulated in HCC tissues compared with that in adjacent paracancer tissues (Figure 1). Ectopic expression of β3GnT8 promoted the metastatic potential of HCC cells *in vitro* and tumorigenesis *in vivo*, whereas knockdown of β3GnT8 inhibited these effects (Figure 2 and 3), suggesting that β3GnT8 is sufficient and essential for HCC initiation and development. Intriguingly, our results also showed that highly expressed β3GnT8 in HCC tissues led to significantly increased levels of polylactosamines compared with the paracancer tissues (Figure 1), which is consistent with the previous report that β3Gn-T8 is involved in the biosynthesis of polylactosamine chains on β1,6-branched *N*-glycans and dramatically upregulated in colon cancer [6]. Based on these findings, it is likely that β3GnT8 promotes HCC initiation and development by altering glycosylation.

Furthermore, our results also showed that the levels of total polylactosamines and glycopattern abundance were significantly elevated in β3GnT8-overexpressing HCC cells, but were markedly reduced in β3GnT8-deficient cells, consistent with previous findings that polylactosamines are synthesized by β3GnT family in which β3GnT8 is a key member [9]. Moreover, β1,6-branches of *N*-linked polylactosamines are commonly observed in malignant transformation and are associated with HCC metastasis [5, 22, 23], whereas inhibition of polylactosamines results in the loss of the metastatic ability [24, 25]. Our results also suggest that β3GnT8 may result in global protein glycosylation (Figure 4), which has been reported as a typical feature in liver cancer [1, 26]. A variety of GTs, including α l,6-fucosyltransferase V, N-acetylglucosamine transferase III and N-acetylglucosamine transferase, have been found highly expressed in tumor tissues or serum of patients with liver cancer, suggesting ubiquitous alteration of glycosylation in HCC cells [5, 27, 28].

Since our previous study has demonstrated that β3GnT8 regulates the metastasis of colorectal cancer cells by altering the glycosylation of cell membrane glycoprotein CD147 [8], we sought to examine the role of β3GnT8 in regulating CD147 glycosylation in HCC cells. Indeed, our results showed that the levels of HG-CD147 in various HCC cell lines were generally elevated following ectopic expression of β3GnT8, and were reduced following knockdown of β3GnT8 (Figure 5), suggesting that β3GnT8 in HCC may be responsible for aberrant glycosylation of CD147. HG-CD147 is highly expressed on the cell surface of a variety of tumor cells [29–31] and can promote metastasis through inducing MMPs, especially MMP-2 and MMP-9, in tumor cells [32]. The studies have revealed that β3GnT8, as an upstream modulator in the CD147 signal transduction pathway [33], regulates glycosylation of CD147, and plays a critical role in tumor metastasis by inducing MMPs expression via Rac1-mediated PI3K/Akt/IKK-dependent IκB-α degradation and NF-κB activation, as well as MKK7/JNK-dependent AP-1 activation [30]. In addition, β3GnT8 may catalyze polylactosamine structures on other glycoproteins, suggesting that β3GnT8 also can affect tumor development through modifying the glycosylation of tumor-associated proteins, and this need to be further investigated.

The present study also revealed that c-Jun is a transcription factor for β3GnT8 and controls β3GnT8 expression in HCC cells. This finding is supported by previous report that c-Jun acts as an oncogene in the liver and strongly promotes liver tumorigenesis in the models of chemical-induced HCC [34, 35]. C-Jun regulates the expression of a number of genes that affect tumor invasion and metastasis by binding to their promoters [36, 37]. It seems that c-Jun/β3GnT8 axis is an important pathway in regulating metastatic potential of HCC cells. However, further investigation is needed to identify novel regulators of β3GnT8, which may provide a better understanding of mechanisms involved in HCC development and progression.

In summary, our study demonstrates that c-Jun-regulated β3GnT8 promotes the metastasis of HCC cells via regulating the N-glycosylation of CD147 and polylactosamines, and provide new insights into function and regulation of β3GnT8 in HCC.

## MATERIALS AND METHODS

### Cell culture

SK-Hep-1, SMMC7721 and HepG2 cell lines were obtained from American Type Culture Collection (Manassas, VA, USA), and were cultured in DMEM supplemented with 10% FBS; (Gibco, Waltham, MA, USA) at 37°C in a humidified atmosphere of 5% CO_2_.

### Experimental animals

Specific pathogen-free male nude mice (aged 5 weeks) were purchased from Shanghai Laboratory Animal Center (Shanghai, China). The mice were housed in specific pathogen-free facilities and all animal studies were approved by the Institutional Laboratory Animal Care and Use Committee of Soochow University.

### Quantitative real-time PCR analysis

Total RNA was extracted from liver cancer cells and reversely transcribed into cDNA using Reverse Transcriptase M-MLV kit (TaKaRa, Japan). Real-time PCR analysis was performed using SYBR Fast qPCR Mix kit (TaKaRa, Japan) and the primers are as follows: GAPDH, 5’-AGAAGGCTGGGGCTCATTTG-3’ (sense) and 5’-AGGGGCCATCCACAGTCTTC-3’ (antisense); β3GnT8, 5’-GTCGCTACAGTGACCTGCTG-3’ (sense) and 5’-GTCTTTGAGCGTCTGGTTGA-3’ (antisense). GAPDH was used as an internal control, and the data were analyzed using the 2^−ΔΔCt^ method.

### Western blot analysis

Cell lysates were harvested and were resolved by SDS-PAGE and transferred to nitrocellulose membranes (EMD Millipore, Billerica, MA, USA). The membranes were blocked with 5% skimmed milk or 1% BSA in TBST at room temperature for 2 h, followed by an overnight incubation with primary antibody against β3GnT8, CD147 (Santa Cruz, Dallas, TX, USA), TIMP2 (Santa Cruz, Dallas, TX, USA), c-jun (Abcam, Cambridge, MA, USA) or GAPDH (Beyotime, Haimen, China) at 4°C. The rabbit anti-human β3GnT8 affinity polyclonal antibody was produced by our laboratory as previously described [20]. Following 3 washes with TBST, the membranes were incubated with HRP-conjugated secondary antibodies (Beyotime, Haimen, China) at room temperature for 1 h. After additional 3 washes with TBST, the protein bands were visualized using an enhanced chemiluminescence (ECL) kit (GE Healthcare Life Sciences, Shanghai, China).

### Immunohistochemistry (IHC) staining and tissue microarray analysis

Tissue microarray slides containing 75 pairs of liver cancer tissue and adjacent paracancer tissue arrays were purchased from Outdo Biotech (Shanghai, China) and stained with a rabbit anti-β3GnT8 antibody and HRP-conjugated anti-rabbit IgG secondary antibody (Beyotime, Haimen, China) or biotinylated-*lycppersicon esculentum* (Tomato) lectin (LEL) and HRP-conjugated streptavidin (Sigama, Sigma, St. Louis, MO, USA). A DAB peroxidase substrate kit (Beyotime, Haimen, China) was used for visualization of enzymatic reaction. The slides were blindly evaluated by an independent pathologist to determine the percentage of positive cells and the staining intensity. Grade 0 denoted positive immunostaining in < 1% cells; 1, 1–33% cells; 2, 34–66% cells; and 3, 67%–100% cells. The staining intensity was quantified as follows: 0, no staining; 1, weak staining; 2, moderate staining; 3, strong staining. The final IHC score was the sum of the grade and the staining intensity: 0, total score = 0; 1+, tot al score = 1–2; 2+, total score = 3–4; 3+, total score = 5–6.

### Plasmids and transfection

β3GnT8 gene was cloned from peripheral blood mononuclear cells (PBMCs) and then inserted into lentiviral vectors expressing yellow fluorescence protein (YFP) [38]. The β3GnT8-expressing-lentiviral vectors were packaged into 293T cells and used to transduce SK-Hep-1 and SMMC7721 cells. The empty vector was used as a negative control. The YFP positive cells were sorted by a FACS Aria III flow cytometer (BD Biosciences, San Jose, CA) to prepare the stable SK-Hep-1 and SMMC7721 cell lines expressing β3GnT8 or vector control.

The plasmids expressing small interfering RNA of β3GnT8 (pSilenCircle-si-β3GnT8), c-jun (pIRES2-EGFR-c-jun), and short hairpin RNA of c-jun (c-jun-shRNA-pGPU6/GFP/Neo) were prepared by our laboratory as previously described [21] or purchased from GenePharma (Suzhou, Jiangsu, China). The empty plasmids pSilenCircle-negative-control, pIRES2-EGFR, and negative-control-shRNA-pGPU6/GFP/Neo were used as negative controls, respectively. Cells were transfected using Liopfectamine 2000 (Invitrogen, Carlsbad, CA, USA) and the following analyses were performed after 48 h of transfection.

### Transwell invasion and migration assays

The invasion and migration assays were performed in 24-well Transwell cell culture chambers (8 μm pore size; Corning, NY, USA) with or without Matrigel (BD Biosciences) precoating, respectively. 1×10^5^ cells resuspended in 200 μL of serum-free DMEM were seeded in the upper chamber and 500 μL of DMEM containing 10% FBS was loaded into the lower chamber. Following 24 h of incubation, the invading and migrating cells in the lower chamber were fixed and stained with eosin staining solution (Beyotime Institute of Biotechnology, Haimen, China). The stained cells were counted in 5 randomly selected fields under a microscope (IX-70, Olympus, Tokyo, Japan) at 200× magnification.

### *In vivo* exnograft tumor model

Five-week-old male nude mice were purchased from Shanghai Laboratory Animal Center (Shanghai, China) and were housed in a specific pathogen-free facility at Soochow University, Jiangsu, China. All animal experiments in this study were approved by the Institutional Laboratory Animal Care and Use Committee of Soochow University. Mice were randomly divided into 2 groups (*n* = 5/group). 1 × 10^6^ SK-Hep-1 or SMMC7721 cells with stable expression of β3GnT8 or vector were inoculated subcutaneously into the right hind leg of each mouse. At 7 days after inoculation, the tumors were measured every 3 or 4 days using a caliper. The tumor volumes were calculated as 1/2×length×width^2^. All mice were sacrificed 21 days after inoculation. Tumors were removed immediately and photographed.

### Co-immunoprecipitation (Co-IP) assay

Cells (SK-hep-1 and HepG2) were lysed with IP lysis buffer as previously described [39]. Cell lysates used for immunoprecipitation was performed with a 1:80 dilution of anti-CD147, anti-β3GnT8 and IgG antibody overnight at 4°C with constant rotation. Antibody-protein conjugates were pulled down by incubation with protein A agarose beads (Thermo Fisher, Rockford, IL, USA) for 4 h at 4°C. Finally, the beads were washed, boiled, centrifuged and the recovered samples were separated by SDS-PAGE for western blot analysis described above. Membranes were detected by both anti-CD147 and anti-β3GnT8 antibodies.

### Lectin blot assay

The protein samples were separated by SDS-PAGE and transferred to nitrocellulose membranes. After blocking with carbo-free blocking solution (Vector Labs, Burlingame, CA, USA), the membranes were incubated with biotinylated-*lycppersicon esculentum* (Tomato) lectin (LEL) for 1 h followed by washes with PBS and additional incubation with HRP-conjugated streptavidin. The blots were then visualized using ECL substrate solution.

### Lectin microarray assay

The lectin microarray was manufactured by Laboratory for Functional Glycomics, College of Life Sciences, Northwest University, China [40]. Lectin microarray assay was performed as previously described [41, 42]. Briefly, 37 lectins (Vector Laboratories, Sigma-Aldrich and Calbiochem) were immobilized on a solid support at a high spatial density. The protein samples isolated from the cells were labeled with Cy3 fluorescence dye (GE Healthcare, Buckinghamshire, UK). The microarrays were scanned using a Genepix 4000B confocal scanner (Axon Instruments, USA). The average background was subtracted and the values less than average background ± 2 were removed from each data point. The median for each lectin was globally normalized to the sum of medians of all valid data for each lectin in one block. Each sample was tested in triplicate and data were presented as the normalized median of each lectin ± standard deviation (SD).

### Matrix-assisted laser desorption/ionization time-of-flight mass spectrometry (MALDI-TOF/TOF-MS) analysis

Proteins (2 mg) from each cell line were denatured with 8 M urea, 10 mM DTT, and 20 mM IAM followed by centrifugation. Samples were washed with 50 mM NH_4_HCO_3_ and desalted by washing with deionized water. The desalted proteins were redissolved with 100 μL of 1M acetohydrazide, 20 μL of 1 M HCl, and 20 μL of 2 M EDC (1-ethyl-3-(3-dimethyllaminopropyl) carbodiimide hydrochloride). The mixture was incubated at room temperature for 4 h to amidate sialylated N-glycans followed by further digestion with PNGase F (New England BioLabs, Ipswich, MA, USA; 1:1000) overnight at 37°C. The released amidated *N*-glycans were collected and lyophilized.

Desalting of *N*-glycans was performed using Sepharose 4B as previously described [43]. Briefly, sepharose 4B in a microtube was washed with methanol/H_2_O (MW; 1:1, v/v) and 1-butanol/methanol/H_2_O (BMW; 5:1:1, v/v/v) under centrifugation. Glycans were dissolved in 500 μL BMW and mixed with sepharose 4B. The mixture was gently shaken for 45 min and washed three times with BMW. *N*-glycans were eluted with MW, collected, and lyophilized.

*N*-glycans were characterized by MALDI-TOF/TOF-MS (UltrafleXtreme, Bruker Daltonics; Bremen, Germany). Lyophilized *N*-glycans were resuspended in 5 μL MW and 1 μL of the suspension was spotted onto an MTP AnchorChip sample target and air-dried. 1 μL of 2,5-dihydroxybenzoic acid (DHB, 20 mg/mL) in MW was spotted to recrystallize the glycans. Mass calibration was performed using peptide calibration standards (250 calibration points; Bruker). Measurements were taken in positive-ion mode and the intense ions from MS spectra were subsequently selected and subjected to MS/MS. Representative MS spectra of *N*-glycans with signal-to-noise ratio > 3 were chosen and annotated using the GlycoWorkbench program with the accuracy < 1.0. Relative intensities were analyzed and generated using a FlexAnalysis software program (Bruker Daltonics). Relative variation was calculated by dividing the relative intensity of a particular type of *N*-glycan by the sum of *N*-glycan relative intensity in one scan, as previously described [44].

### Chromatin immunoprecipitation (ChIP) assay

ChIP was performed using a ChIP assay kit (Beyotime, Haimen, China) according to the manufacturer's protocol. Chromatin solutions were sonicated and incubated with the anti-c-Jun antibody (Abcam, Cambridge, MA, USA) or mouse control IgG (Beyotime, Haimen, China) while rotating overnight at 4°C. The solution was washed according to the manufacturer's instruction. DNA-protein cross-links were reversed, and chromatin DNA was purified and subjected to PCR analysis using the Easy-Load PCR Master mix (Beyotime, Haimen, China). Primers 5’-TGTACGCGTGAGGCACATGGCAAAGG-3’ (forward) and 5’-GTTCTCGAGAGTGGGGAGGAAGTGGT-3’ (reverse) were used to amplify the β3GnT8 promoter sequence [19]. Following amplification, PCR products were resolved on a 1.5% agarose gel and visualized by ethidium bromide staining.

### Statistical analysis

Statistical analysis was performed using SPSS software 22.0 (IBM SPSS, Armonk, NY, USA). Results are presented as the mean ± SD. Student's *t*-test was used to evaluate the significance of data. *P* < 0.05 was considered statistically significant.

## SUPPLEMENTARY MATERIALS FIGURES AND TABLES


