# Supplementary material for: c-Jun-dependent β3GnT8 promotes tumorigenesis and metastasis of hepatocellular carcinoma by inducing CD147 glycosylation and altering N-glycan patterns
Source: Oncotarget. 2018 Jan 12;9(26):18327–40. doi: 10.18632/oncotarget.24192 (PMC5915075; doi:10.18632/oncotarget.24192)
Supplement: Supplementary file 1 [file oncotarget-09-18327-s001.pdf]

c-Jun-dependent β3GnT8 promotes tumorigenesis and metastasis of hepatocellular carcinoma by inducing CD147 glycosylation and altering *N*-glycan patterns

SUPPLEMENTARY MATERIALS

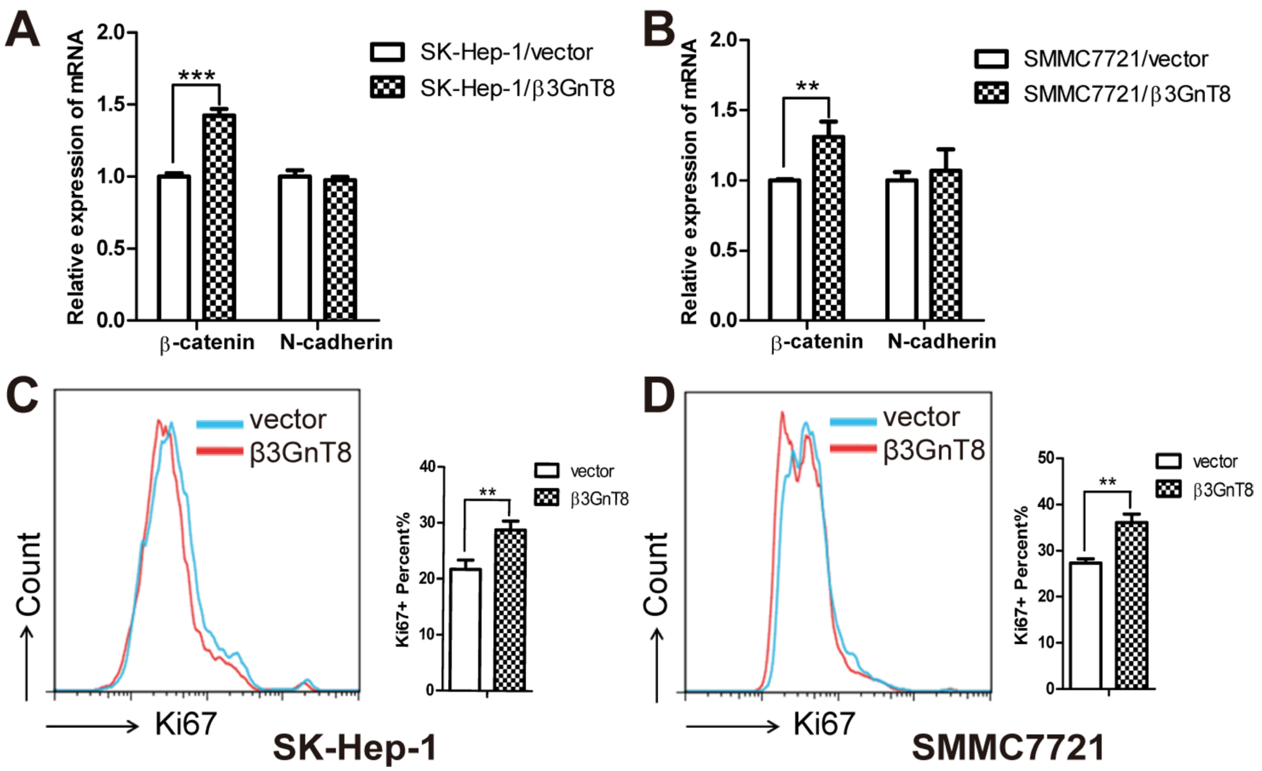

Supplementary Figure 1: The effects of β3GnT8 on the expression of EMT and proliferation markers in HCC cells. (**A**) Quantitative RT-PCR analysis of β-catenin and N-cadherin expression in Sk-Hep-1/vector and SK-Hep-1/β3GnT8 cells. (**B**) Quantitative RT-PCR analysis of β-catenin and N-cadherin expression in SMMC7721/vector and SMMC7721/β3GnT8 cells. (**C**) Flow cytometric analysis of the percentages of Ki67+ cells in Sk-Hep-1/vector and SK-Hep-1/β3GnT8 cells. (**D**) Flow cytometric analysis of the percentages of Ki67+ cells in SMMC7721/vector and SMMC7721/β3GnT8 cells. Data are representative of three independent experiments and presented as means ± SD; $^{*}p < 0.05$, $^{**}p < 0.01$, $^{***}p < 0.001$.

Supplementary Table 1: Proposed structures and their molecular ions in MALDI spectra of N-glycans in SK-Hep-1/vector and SK-Hep-1/β3GnT8 cell lines

No.	Observed [M+Na]+	Relative Intensity		Glycan Structure
		SK-Hep-1/vector	SK-Hep-1/β3GnT8	
1	1080.347124	0.0421 ± 0.0073	0.0234 ± 0.0037	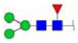
2	1096.340198	0.0032 ± 0.0029	0.0019 ± 0.0016	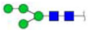
3	1241.839062	0.0040 ± 0.0051	0.0039 ± 0.0003	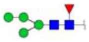
4	1258.389906	0.0418 ± 0.0044	0.0264 ± 0.0066	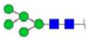
5	1299.429848	--	0.0035 ± 0.0002	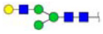
6	1403.626705	--	0.0036 ± 0.0003	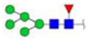
7	1420.439541	0.2672 ± 0.0244	0.2026 ± 0.0110	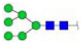
8	1445.481473	0.0097 ± 0.0004	0.0089 ± 0.0002	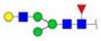
9	1486.519991	0.0038 ± 0.0033	0.0046 ± 0.0003	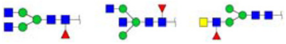
10	1565.493635	0.0041 ± 0.0038	0.0067 ± 0.0005	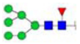
11	1582.491929	0.1684 ± 0.0055	0.1620 ± 0.0104	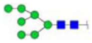
12	1664.540566	0.0197 ± 0.0035	0.0250 ± 0.0046	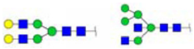
13	1727.169618	--	0.0055 ± 0.0008	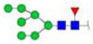
14	1744.544409	0.1944 ± 0.0055	0.2016 ± 0.0081	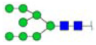
	Or 1745.6345	0.1944 ± 0.0055	0.2016 ± 0.0081	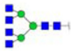
15	1810.612449	0.0305 ± 0.0063	0.0398 ± 0.0094	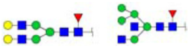
16	1867.154849	--	0.0016 ± 0.0014	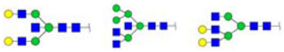
17	1906.591521	0.1792 ± 0.0090	0.2140 ± 0.0138	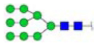
18	2029.683418	0.0084 ± 0.0028	0.0179 ± 0.0067	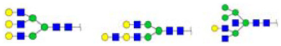
19	2175.745972	0.0134 ± 0.0045	0.0240 ± 0.0092	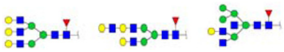
20	2394.80941	0.0026 ± 0.0027	0.0063 ± 0.0026	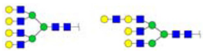
21	2540.872811	0.0077 ± 0.0040	0.0168 ± 0.0071	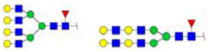

N-Acetylglucosamine (GlcNAc)   Mannose (Man)   Galactose (Gal)   Fucose (Fuc)

N-Acetylgalactossamine (GalNAc)

N-glycans with SD were identified in three independent experiments.
